# Free Radical Production, Inflammation and Apoptosis in Patients Treated With Titanium Mandibular Fixations—An Observational Study

**DOI:** 10.3389/fimmu.2019.02662

**Published:** 2019-11-08

**Authors:** Jan Borys, Mateusz Maciejczyk, Bożena Antonowicz, Jarosław Sidun, Magdalena Świderska, Anna Zalewska

**Affiliations:** ^1^Department of Maxillofacial and Plastic Surgery, Medical University of Bialystok, Bialystok, Poland; ^2^Department of Hygiene, Epidemiology and Ergonomics, Medical University of Bialystok, Bialystok, Poland; ^3^Department of Oral Surgery, Medical University of Bialystok, Bialystok, Poland; ^4^Department of Material and Biomedical Engineering, Faculty of Mechanical Engineering, Bialystok University of Technology, Bialystok, Poland; ^5^Experimental Dentistry Laboratory, Medical University of Bialystok, Bialystok, Poland

**Keywords:** titanium implants, mandible fracture, inflammation, free radicals, apoptosis

## Abstract

Despite high biocompatibility of titanium and its alloys, this metal causes various side effects in the human body. It is believed that titanium biomaterials may induce an innate/adaptive immune response. However, still little is known about changes caused by titanium mandible implants, particularly with regard to bone healing. The latest studies showed disturbances in the antioxidant barrier, increased oxidative/nitrosative stress, as well as mitochondrial abnormalities in the periosteum covering titanium mandible fixations; nevertheless, the impact of titanium implants on free radical production, inflammation, and mandible apoptosis are still unknown. Because severe inflammation and apoptosis are among the main factors responsible for disturbances in osteointegration as well as implant rejection, this study is the first to evaluate pro-oxidant enzymes, cytokines as well as pro- and anti-apoptotic proteins in the periosteum of patients with a broken jaw, treated with titanium miniplates and miniscrews. The study group consisted of 29 patients with double-sided fracture of the mandible body requiring surgical treatment. We found significantly higher activity of NADPH oxidase and xanthine oxidase as well as enhanced rate of free radical production in the periosteum of patients in the study group compared to the control group. The markers of inflammation [interleukin 1 (IL-1), interleukin 6 (IL-6), tumor necrosis factor α (TNF-α), transforming growth factor β (TGF-β) and β-glucuronidase (GLU)] as well as apoptosis [Bax, Bax/Bcl-2 ratio, caspase-3 (CAS-3) and nitric oxide (NO)] were significantly elevated in periosteum covering titanium fixations compared to the control group. In the study group, we also demonstrated an increased content of titanium on the periosteum surface, which positively correlated with CAS-3 activity. The study led us to the conclusion that titanium mandible implants increase the production of pro-inflammatory cytokines, and enhance free radical generation in the periosteum covering titanium miniplates and miniscrews. Additionally, exposure to Ti6Al4V titanium alloy induces apoptosis in the mandible periosteum. However, no clinical signs of the said phenomena have been observed.

## Introduction

Titanium miniplates and miniscrews are widely used in the surgical treatment of jawbone fractures. However, the studies by Rosenberg et al. (1), Schliephake et al. (2), and Jorgenson et al. (3) already showed that titanium jawbone fixation devices corrode in the body environment, and their wear debris was found in the tissues covering the miniplates as well as in the nearby lymph nodes. In the absence of thorough data on the long-term effects of titanium fixations and their wear debris on the body, the grounds of removing miniplates and miniscrews after the fracture healing are questionable (4–7). Interestingly, in the 1970s, the removal of miniplates and miniscrews used as a kind of rigid internal fixation was a standard procedure (8, 9). Nowadays, jawbone fixations are most often removed only in case of various complications, such as infections, pain, metal toxicity, allergy, growth disturbance, plate migration, palpability, thermal sensitivity, as well as planned implant treatment or difficulties in CT and MR medical imaging (4, 5, 9, 10). It is estimated that the percentage of patients in whom jawbone fixations were removed after osteosynthesis ranged from 9 to 24.6% (5, 7, 10).

Titanium implant wear debris is not biologically indifferent to the body (11–14). Indeed, numerous studies indicate that the passive layer of titanium dioxide (TiO_2_) on the surface of the implant may be damaged by mechanical friction and/or electrochemical corrosion, which leads to the transfer of metallic particles to the surrounding tissues, and their accumulation (3, 11, 15, 16). Moreover, *in vitro* studies have shown that implant wear products can cause macrophage activation and inflammation, resulting in the production of large quantities of pro-inflammatory cytokines and reactive oxygen species (ROS) (11, 12, 17, 18). Interestingly, aseptic chronic inflammation occurring as a reaction to implant wear debris may also induce osteolysis around the implant and lead to its loss (19). It has been demonstrated that particles and ions of titanium induce genomic instability in human fibroblast cells (20), inhibit the synthesis of type I collagen by osteoblasts (21) and stimulate the production of titanium (IV)-specific T-lymphocytes (22). These conclusions are supported by the observed presence of dark discoloration of the tissues adjacent to the surface of titanium implants, and accumulation of inflammatory cells (macrophages, T and B lymphocytes) in tissues around the removed long bone implants in patients (16). It is suggested that transglutaminase 2, calcium-dependent enzyme, plays a critical role in the early phase of inflammation associated with nuclear factor-kappa B (NF-κB) activation and alterations in RANKL (receptor activator of nuclear factor-kappa B ligand)/ OPG (osteoprotegerin) homeostasis (23). It is well-known that RANKL induces bone resorption by osteoclasts, and RANKL/OPG imbalances are involved in the pathogenesis of periodontitis as well as bone/mineral metabolism disorders (23, 24). The results of recent studies also indicate that the wettability of titanium surfaces may be responsible for the speed of implant osteointegration (25). Interestingly, it has been demonstrated that shifts in macrophage polarization from a pro-inflammatory (M1, classically activated macrophages) to an anti-inflammatory (M2, alternatively activated macrophages) state may improve implant integration (25, 26). Therefore, *in vitro* basic research may allow increasing the biocompatibility of titanium implants.

In our earlier studies, we demonstrated disturbances in cellular redox homeostasis, increased oxidative/nitrosative stress, and mitochondrial dysfunction in the periosteum covering titanium mandible fixations (27–29). However, the impact of titanium implants on free radical formation, cytokine expression, as well as cellular apoptosis are still unknown in patients treated with mandible fixations. It is well-known that the balance between the number of cells, their differentiation and apoptosis plays a key role in bone metabolism; nevertheless, many exogenous factors (including titanium) can interfere with bone formation/resorption, and therefore, impair the bone healing. Interestingly, it was shown that increased inflammation and apoptosis are responsible for alterations in osteointegration as well as implant rejection (30). Although *in vitro* studies have revealed that titanium wear debris induce osteoblast apoptosis and enhance pro-inflammatory cell-mediated immune response (31), still little is known about the changes caused by titanium implants in the mandible and jaw, particularly in terms of bone healing. Bearing in mind that mitochondrial abnormalities and ROS overproduction are factors inducing apoptosis and may initiate/intensify inflammation, our study aims to evaluate pro-oxidant enzymes, cytokines as well as pro- and anti-apoptotic proteins in the periosteum of patients with a broken jaw, treated with titanium miniplates and miniscrews.

## Materials and Methods

### Ethical Issues

The experiment was conducted in accordance with the Declaration of Helsinki (1964). All patients were informed about the purpose of the study and type of the planned examinations, and signed a conscious consent to participate in the experiment. The protocol of the study was approved by the Bioethics Committee of the Medical University of Bialystok, Poland (permission number R-I-002/3/2-16).

### Patients

Fifty eight patients treated at the Department of Maxillofacial and Plastic Surgery, Medical University of Bialystok, Poland were recruited to participate in this study. On admission to hospital, all patients had computed tomography (CT) and blood tests performed. Only healthy individuals with normal body weight (18.5 ≤ BMI ≥ 24.5) and without any systemic diseases, such as cardiovascular diseases, cancer, thyroid, lung, or kidney diseases as well as type 1 and type 2 diabetes were included in the experiment. The number of patients was set based on a previously conducted pilot study. The power of the test was set at 0.9.

The study group consisted of 29 patients (6 women and 23 men), aged 19–29 (mean age: 23 years and 5 months), with double-sided fracture of the mandible body requiring surgical treatment. The causes of the fractures in patients were: beating (55.2%), sports (24.5%), traffic accidents (12.5%), unfortunate falls (4.5%), and accidents at work (3.3%). Individuals with either single or multiple fracture of the mandible as well as fracture of mandibular ramus or condyle were excluded from the study. Fractures of the mandible were treated with lamellar osteosynthesis by means of titanium miniplates and miniscrews after setting the fracture. Each patient had two 4- or 6-eyelet plates inserted with 16–24 screws (Ti6Al4V titanium alloy; MEDGAL Sp. z o.o., Ksiezyno, Poland).

The control group consisted of 29 patients matched by age and gender with the study group (6 women and 23 men; mean age; 22 years and 4 months), in whom we removed the incorrectly placed, completely impacted mandibular third molars included in III C mesioangular (20) and vertical (9) position according to the Pell and Gregory (32) as well as Winter (33) classifications. No patient from the control group reported any pain, and their tooth retentions were not complicated by inflammation. The teeth were removed only based on orthodontic recommendations.

The criteria excluding patients from the control and study group were: inflammatory complications and jaw synostosis disorders after implantation, wounds of soft tissues, injury of the skull, chest, abdomen, and extremities, fracture of other bones, as well as operations due to bone fractures in the past. We excluded patients with chronic systemic diseases such as: cancers, metabolic diseases (obesity, hypertension, insulin resistance, diabetes), cardiovascular diseases (ischaemic heart disease, atherosclerosis, conductivity disorders), autoimmune diseases (Sjögren syndrome, scleroderma sclerosis, rheumatoid arthritis), gastrointestinal tract, thyroid, lung and infectious diseases (HIV, HCV, HBV). Smokers, alcoholics, patients with oral diseases, periodontitis [probing pocket depth (PPD) > 2; bleeding on probing (BOP) > 15] or active odontogenic infection foci were also eliminated from the study. Moreover, the experiment did not involve persons taking any medicines, e.g., antibiotics, glucocorticoids, non-steroidal anti-inflammatory drugs, and dietary supplements, vitamins, microelements (preparations of iron, selenium, zinc, iodine) within 3 months from the beginning of the study.

### Experimental Procedures

Immediately after the procedure and within 3–5 months until the removal of titanium plates, all patients had been fed a balanced diet (2,200 kcal; 55% carbohydrates, 30% fat, and 15% protein) developed by a dietitian. After the end of hospitalization, every 2 weeks the patients reported for follow-up visits to an experienced maxillo-facial surgery specialist (JB). Throughout the entire period after the procedure, no symptoms of acute and chronic inflammation, such as reddening, oedema, inflammatory infiltration, abscess, or purulent fistula were observed in physical examinations in the implant site. No enlargement of the surrounding lymph nodes or allergy symptoms, such as oedema, changes on the skin, and oral mucosa were found. After 3–5 months after osteosynthesis, the titanium fixations of the mandible were removed at the explicit request of the patient. The most common reasons for the removal of miniplates and miniscrews were: planned implant-prosthetic treatment after tooth loss (18 patients) and discomfort connected with palpably felt fixation (5 patients). Three people reported hypersensitivity during the consumption of hot or cold food. In other patients, titanium fixations were removed as a precautionary measure in order to avoid potential risk of allergic reactions to foreign bodies in the future.

### Collection of Periosteum Samples

In the study group, the periosteum was collected during the removal of titanium fixations (miniplates and miniscrews) 3–5 months after osteosynthesis. The procedure was performed under local anesthesia of 2% lignocaine with adrenaline (Polfa, Warsaw, Poland) by the same surgeon (JB). The research material consisted of small fragments (sized 3 × 7 mm and about 1 mm thick) of gray-pigmented tissue adhering to the implants excised during the removal of the mandible fixations.

In the control group, the study required the collection of healthy periosteum from the oblique line area after incision and separation of the mucoperiosteal flap during impacted mandibular third molars exposition and extraction.

The collected tissues were immediately immersed in liquid nitrogen and then placed in the temperature of −80°C. They were stored in the said conditions until the assays were performed (but not longer than 6 months).

### Preparation of Periosteum Homogenates

On the day of the assays, the tissue samples were slowly thawed at +4°C, weighed and fragmented with surgical scissors. The prepared tissue was transferred to glass beakers placed in ice and containing ice-cold phosphate-buffered saline (PBS) in the ratio of 1 gram of tissue per 9 mL of PBS. In order to protect the samples against oxidation and proteolysis processes, 0.5 M BHT (butylated hydroxytoluene; 10 μL/1 mL PBS) and protease inhibitor (1 tablet/10 mL PBS; Complete Mini, Roche, France) were added to the homogenates (34). The tissues were homogenized with a knife homogenizer (Omni TH, Omni International, Kennesaw, GA, USA) at a speed of 5,000 rpm (the beaker was kept in ice bath) (35). The obtained tissue suspensions were sonified in ice bath (1,800 J/sample, 3 × 20 s; UP 400S sonicator, Hielscher, Teltow, Germany) and then centrifuged for 10 min at 5,000 × g at 4°C (35). The supernatant liquid was preserved for further research and was immediately used for the assays.

### Determination of Pro-oxidant Enzymes

The activity of NADPH oxidase (NOX, EC 1.6.3.1), xanthine oxidase (XO, EC 1.17.3.2), and the rate of ROS production were measured immediately after periosteum collection (36). NOX activity was determined by luminescence assay using lucigenin as an electron acceptor (37). One unit of NOX activity was defined as the amount of enzyme required to release 1 nmol of the superoxide anion per 1 min. XO activity was determined colorimetrically by measuring the increase in uric acid (UA) absorbance at 290 nm (38). One unit of XO activity was defined as the amount of enzyme required to release 1 μmol of UA per 1 min. The rate of free radical production was determined fluorimetrically using 2,7-dichlorodihydrofluorescein diacetate (DCFH-DA), which is de-esterified to 2,7-dichlorodihydrofluorescein (DCFH) by ROS (39). ROS production rate was calculated from the calibration curve for DCFH.

### Determination of Inflammation

The concentration of interleukin 1 (IL-1), interleukin 6 (IL-6), tumor necrosis factor α (TNF-α) and transforming growth factor β (TGF-β) was determined with the use of commercial ELISA kits (Human Interleukin 1 alpha, IL-1 alpha ELISA Kit, EIAab; Human Interleukin 6, IL-6 ELISA Kit, EIAab; Human Tumor necrosis factor ELISA Kit, EIAab; Human Transforming growth factor beta-1, TGF-beta-1 ELISA Kit, EIAab) according to the manufacturer's instructions. The activity of β-glucuronidase (GLU, EC 3.2.1.31) was estimated colorimetrically using 4-nitrophenyl-β-D-glucuronide as a substrate (40). The intensity of the released 4-nitrophenol was measured at 405 nm wavelength.

### Determination of Apoptosis

The concentration of nitric oxide (NO) was assayed by the Griess method based on the reaction of nitrates with sulfanilamide and N-(1-naphthyl)-ethylenediamine dihydrochloride, resulting in a colored product with a maximum absorbance at 490 nm wavelength (41). The activity of caspase-3 (CAS-3) was measured colorimetrically with Ac-Asp-Glu-Val-Asp-p-nitroanilide as a substrate (42). The quantity of p-nitroaniline (pNA) released by CAS-3 activity was measured at 405 nm wavelength. The concentration of Bax and Bcl-2 in the periosteum of the study as well as the control group was determined by means of commercial ELISA kits (Human Apoptosis regulator BAX ELISA Kit, EIAab; Human Bcl-2 ELISA Kit, EIAab), according to the manufacturer's recommendations.

### Determination of Total Protein

The concentration of total protein was determined with the bicinchoninic acid (BCA) method (43) with bovine serum albumin (BSA) as a standard. A commercial kit (Thermo Scientific PIERCE BCA Protein Assay) was used according to the manufacturer's instructions.

### Determination of Metallosis

Metallosis was determined based on Energy Dispersive X-Ray Spectroscopy (EDS) (29). For this purpose, a periosteum sample with the dimensions of 2 × 2 mm was collected from the former fracture site at the implants, at the projection of the first screw. The composition of elements was measured in the center and in two points diagonally, approximately 0.5 mm from the center of the periosteum fragment. In the experiment, Hitachi S-3000N electron scanning microscope equipped with NSS X-ray spectrometer (Noran System Six) and a freezing table for biological specimens were used. The surface of the plates was also viewed under an optical microscope directly before implantation and immediately after periosteum extraction.

### Histological Examination

Immediately after collection, fragments of periosteum were placed in 10% buffered formalin. The tissues were immersed in paraffin cubes which were then cut into 10-μm-thick pieces. The histological preparations were stained with haematoxylin and eosin (HE), and evaluated under an optical microscope at a magnification of 40 x.

### Statistical Analysis

Statistical analysis was performed using the GraphPad Prism for MacOS (GraphPad Software, La Jolla, USA). To confirm the normal distribution of the results, D'Agostino-Pearson test and Shapiro-Wilk test were used. The Student's *t*-test was performed to compare the results from the study and the control group. In the lack of normal distribution of the results, the Mann–Whitney *U* test was used. The correlations between the measured parameters were tested by the Pearson correlation coefficient. All data was presented as the mean ± SEM. The statistical significance was defined as *p* ≤ 0.05.

## Results

### Clinical Characteristics

Clinical characteristics of patients are presented in Table 1. No significant differences in blood morphology and biochemical parameters were observed in patients from the study group and the control group.

**Table 1:** Clinical characteristics of patients.

	**Control group** ***N* = 30**	**Study group** ***N* = 30**
Mean age	22 years 4 months	23 years 5 months
Male/Female	23/6	23/6
WBC (×10^3^/μL)	7.25 ± 0.50	7.07 ± 0.46
RBC (×10^6^/μL)	5.14 ± 0.10	5.17 ± 0.09
HGB (g/dL)	15.29 ± 0.24	15.28 ± 0.21
HCT (%)	45.38 ± 0.64	45.63 ± 0.58
MCV (fL)	88.29 ± 1.05	87.88 ± 1.06
MCHC (g/dL)	33.71 ± 0.33	33.44 ± 0.34
PLT (×10^3^/μL)	229.60 ± 9.56	231.30 ± 8.73
PT (s)	11.96 ± 0.19	12.04 ± 0.24
Ratio PT (%)	110.90 ± 2.75	107.9 ± 3.69
INR	0.93 ± 0.02	0.94 ± 0.03
APTT (s)	27.46 ± 0.71	27.68 ± 0.55
Fibrinogen (mg/dL)	263.10 ± 13.79	267.1 ± 10.19
Na^+^ (mmol/L)	140.70 ± 0.62	140.4 ± 0.40
K^+^ (mmol/L)	4.37 ± 0.08	4.37 ± 0.06
CRP (mg/L)	1.12 ± 0.14	1.19 ± 0.14
AST (IU/L)	32.00 ± 2.12	32.73 ± 1.79
ALT (IU/L)	32.90 ± 1.76	29.47 ± 1.42

*WBC, White Blood Cells; RBC, Red Blood Cells; HGB, Hemoglobin; HCT, Hematocrit; MCV, Mean Corpuscular Volume; MCHC, Mean Corpuscular Hemoglobin Concentration; PLT, Platelets; PT, Prothrombin Time; Ratio PT, Ratio Prothrombin Time; INR, International Normalized Ratio; APTT, Activated Partial Thromboplastin Time; Na^+^, Sodium Ion; K^+^, Potassium Ion; CRP, C-Reactive Protein; AST, Aspartate Transaminase; ALT, Alanine Transaminase*.

### Pro-oxidant Enzymes

**Figure 1:**
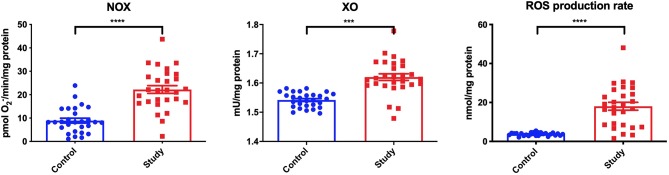
Pro-oxidant enzymes [NADPH oxidase (NOX) and xanthine oxidase (XO)] and reactive oxygen species (ROS) production in the periosteum of the study and the control group. ***p < 0.0005, ****p < 0.0001.

In the study group, significantly higher activity of NOX (+187.5%) was observed in the homogenates of periosteum covering titanium mandible fixations, compared to the control group. Similarly, the activity of XO in the mandibular periosteum was significantly higher in the study group than in the control group. In periosteum homogenates in the study group a significantly higher ROS production (+533.3%) was also found, compared to controls (Figure 1).

### Inflammation

**Figure 2:**
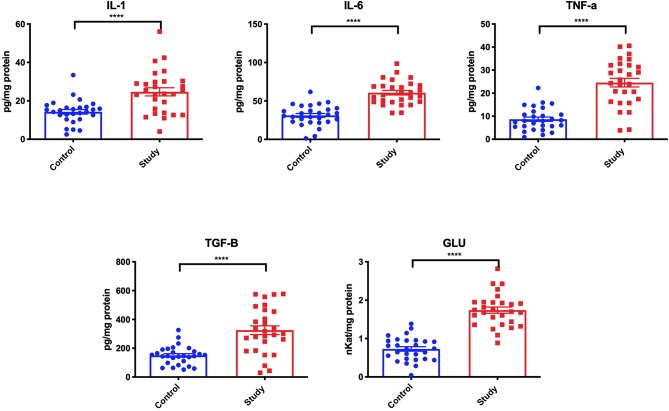
Inflammatory response [interleukin 1 (IL-1), interleukin 6 (IL-6), tumor necrosis factor α (TNF-α), transforming growth factor β (TGF-β) and β-glucuronidase (GLU)] in the periosteum of the study and the control group. ****p < 0.0001.

Significantly higher concentrations of IL-1 (+100%), IL-6 (+100%), TNF-α (+200%), and TGF-β (+40%), as well as significantly higher GLU activity (+130%) were demonstrated in the homogenates of periosteum covering titanium mandible fixations, compared to the control group (Figure 2).

### Apoptosis

**Figure 3:**
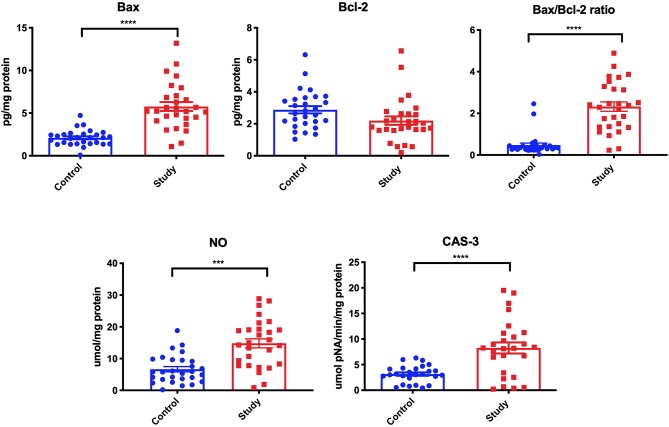
The concentration of Bax, Bcl-2, Bax/Bcl-2 ratio, nitric oxide (NO), and activity of caspase-3 (CAS-3) in the periosteum of the study and the control group. ***p < 0.0005, ****p < 0.0001.

Bax concentration in the mandibular periosteum of patients from the study group was considerably higher (+161.1%) than in the control group. In these patients, a significantly higher Bax/Bcl-2 ratio (+301.7%) was also observed compared to the control group, even though the concentration of Bcl-2 did not differ much. Considerably higher concentration of NO (+115.4%) in periosteum homogenates of patients was observed in the study group compared to the controls. Similarly, CAS-3 activity in the mandibular periosteum around miniplates and miniscrews was significantly higher in the study group (+196.3%) compared to the control group (Figure 3).

### Metallosis

**Figure 4:**
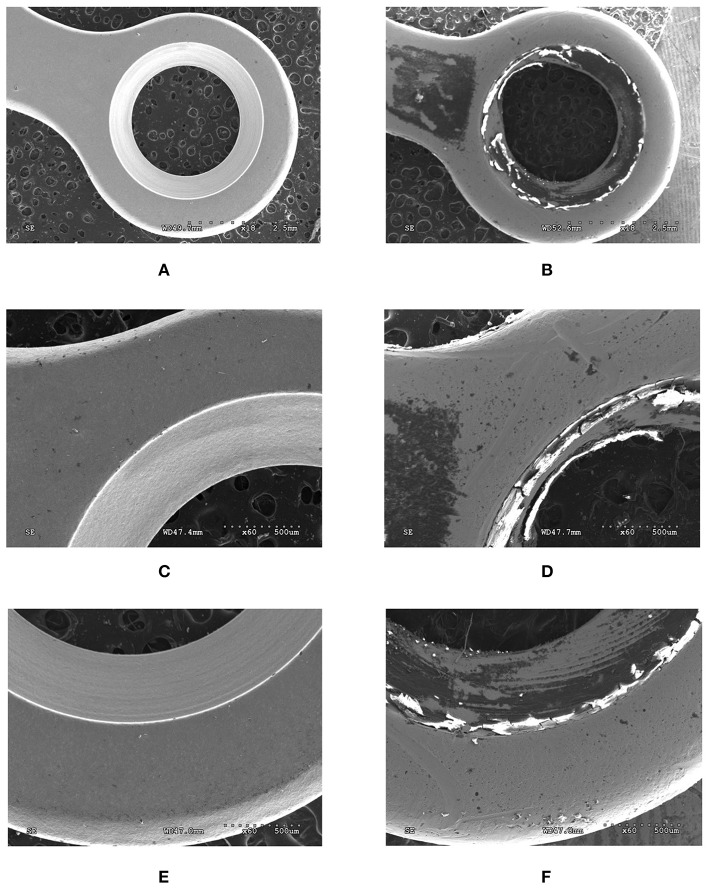
View of the surface of titanium miniplates before **(A,C,E)** and after **(B,D,F)** implantation. Magnification × 18 **(A,B)**, × 60 **(C–F)**.

The condition of miniplates before and after implantation is presented in Figure 4. It is clearly visible that the surface of the miniplate after its removal is entirely different from that before implantation. The presented damage is plastic deformation that may result from the impact of the hole and screw, and the act of the miniplate assembly.

**Figure 5:**
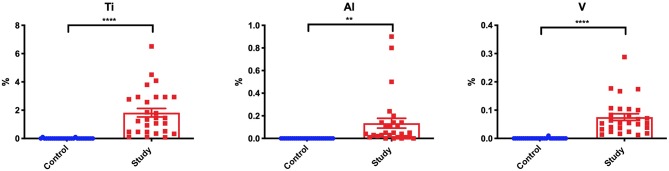
Content of titanium (Ti), aluminum (Al), and vanadium (V) on the periosteum surface in the study and the control group. **p < 0.005, ****p < 0.0001.

EDS analysis showed a significantly higher level of titanium (Ti), aluminum (Al), and vanadium (V) on the periosteum surface in patients treated with titanium mandible implants compared to control group (Figure 5).

### Histological Examination

**Figure 6:**
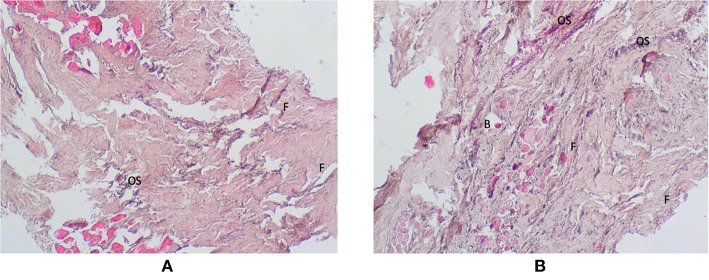
Histological examination of the periosteum tissue of the control **(A)** and the study group **(B)**. Magnification × 40. B, blood vessels; F, fibroblasts; OS, osteoblasts.

In the periosteum of the study group patients, we observed a small inflow of inflammatory cells as well as a greater number of fibroblasts and osteoblasts in connective tissue compared to the controls (Figure 6).

### Correlations

**Figure 7:**
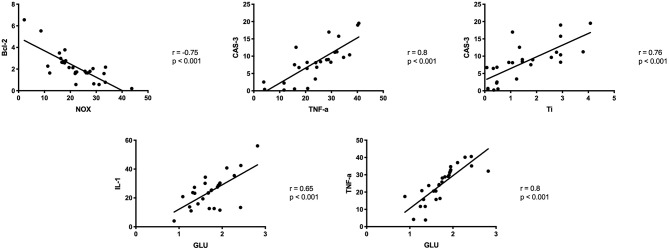
Correlations between ROS production, inflammation and apoptosis biomarkers in the periosteum of the study group. CAS-3, caspase 3; GLU, β-glucuronidase; IL-1, Interleukin 1; IL-6, interleukin 6, Ti, titanium; TNF-α, tumor necrosis factor α.

In the periosteum of patients from the study group, a negative correlation was observed between NOX activity and Bcl-2 concentration, as well as positive correlation between Bax concentration and CAS-3 activity, TNF-α concentration and CAS-3 activity, and the titanium content and CAS-3 activity. There was also a positive correlation between the activity of GLU and the concentration of proinflammatory cytokines: IL-1 and TNF-α (Figure 7).

## Discussion

In earlier studies, we demonstrated increased oxidative stress and mitochondrial dysfunction in the periosteum of patients treated with titanium mandibular implants (27–29). The presented study is the first to indicate increased production of oxygen free radicals as well as ROS-induced inflammation and apoptosis in the tissue surrounding titanium fixations, which may disturb bone healing during the fracture.

Numerous experimental and clinical studies have shown increased release of metal ions from the implant surface both into the surrounding tissues (44) and extracellular space (45). In our study, we also observed a significantly higher content of titanium, aluminum and vanadium in the periosteum covering titanium fixations in all patients from the study group (Figure 5). Thus, our results confirm metallosis in patients treated with mandible implants. These metals are derived from wear and tear processes (corrosion) observed on the surface of miniplates and miniscrews (Figure 4). The corrosive phenomena may be caused by friction between metal against metal, which increases the tribological wear and leads to fatigue destruction of the biomaterials of which the fixations are made. Increased release of particles or titanium ions can also occur during implantation. Haynes and the co-authors (14) draw attention to the correlation between the presence of metallic particles released from titanium implants and increased production of inflammatory mediators and bone resorption markers. *In vitro* studies have proven that contact with TiALV titanium alloy leads to increased release of prostaglandin E2 (PGE2), IL-1, IL-6, and TNF-α (14). It is believed that similar phenomena may also occur in the fixations of jaw bones, as corrosive friction between screws and a miniplate as well as electrochemical corrosion of the implant in the environment of tissue fluids have been observed in patients with mandible fractures (6, 27).

In the presented study we demonstrated significantly higher concentrations of IL-1, IL-6, TNF-α and TGF-β as well as considerably higher activity of β-glucuronidase in the mandibular periosteum covering titanium fixations. Raised concentration of pro-inflammatory cytokines may indicate inflammation around titanium implants. It is highly probable that products of titanium miniplates and miniscrews degradation stimulate macrophages, fibroblasts and T lymphocytes to produce pro-inflammatory cytokines as well as proteolytic enzymes from the group of lysosomal exoglycosidases (13, 21). TNF-α is a key cytokine responsible for aseptic bone atrophy around implants (46). TNF-α has been shown to intensify inflammation, induce differentiation of preosteoclasts, increase the pool of osteoclast precursors, and promote the survival of osteoclasts by increasing their activity (30, 46). TNF-α also increases RANKL expression in osteoblasts and bone marrow cells (46). Zreiqat et al. (47) also demonstrated that IL-1, IL-6, TNF-α, and PGE2 modify the expression of thrombospondin, alkaline phosphatase, osteocalcin, osteopontin, and mRNA for procollagen I. These cytokines are therefore important factors regulating the activity of human bone-derived cells. The results of the said studies support the hypothesis that titanium wear debris modulates the activity of osteoblasts by changing the gene profile and affecting bone formation and mineralization (47). Interestingly, recent data indicated that the production of pro-inflammatory cytokines may depend on the enhanced transglutaminase 2 expression, especially in the initial stage of bone healing and inflammation (23). Indeed, exposure to orthodontic implants has been shown to promote both IL-6 and transglutaminase 2 release in a human gingival fibroblast cell line (48). Transglutaminase 2 can also promote NF-κB activation, while the loss in its activity results in decreased expression of NF-κB and RANKL (23). Thus, transglutaminase 2 may play a key role in the balance between bone remodeling and resorption.

Titanium ions released from implants stimulate macrophages and osteoclasts to increase the production of not only pro-inflammatory cytokines, but also ROS and reactive nitrogen species (RNS) (13, 18, 21). The main source of ROS/RNS in cells are mitochondria and pro-oxidant enzymes: NADPH oxidase (NOX) and xanthine oxidase (XO) (18, 49). Our earlier studies revealed that disturbances in the activity of the mandible periosteum mitochondria may induce oxidative and nitrosative stress (29). In the study presented herein, we were the first to demonstrate significantly higher activity of pro-oxidant enzymes: NOX (+187.5%) and XO, as well as a considerably higher rate of ROS production (+533.3%) in the periosteum covering titanium miniplates and miniscrews compared to the controls. It is believed that excessive production of ROS leads to mitochondrial damage resulting in overproduction of oxygen free radicals by the so-called ROS-induced ROS release (49, 50). Importantly, increased ROS production may also boost the release of titanium ions from the surface of implants through ROS-induced corrosion. This condition intensifies inflammatory processes and leads to dysfunction of vascular endothelium and angiogenesis in the bone adjacent to titanium implants (51). Moreover, increased production of proinflammatory cytokines may be responsible for the formation of more free radicals (by the principle of positive feedback), which initiates cell death by apoptosis. In our study, we also observed increased activity of β-glucuronidase, which is a lysosomal enzyme released from granular leukocytes. It is assumed that oxidative stress is responsible for increasing the release of GLU from lysosomes (40). Oxidative damage to proteins and lipids of lysosomal membranes leads to membrane cracking, resulting in the release of lysosomal enzymes involved in cell apoptosis into the cytoplasm. Interestingly, in our study we noted a positive correlation between GLU activity and IL-1 and TNF-α levels, which may indicate pro-inflammatory influence of GLU when exposed to Ti4Al4V titanium alloy. This is also confirmed by the results of histological examinations in which we demonstrated increased inflammatory reaction (inflow of leukocytes) in the periosteum surrounding titanium implants (Figure 6). However, it should be noted that increased GLU activity and higher level of TGF-β (bone formation marker) in study group patients may indicate not only the induction of local inflammation, but also increased bone resorption or mandible remodeling during fracture healing (52–54). Indeed, GLU catalyzes the hydrolysis of glucuronic acid residues from glycoproteins and bone proteoglycans, which occurs during bone remodeling in the process of fracture healing (54, 55). Thereby, this issue requires further research and clinical observations. Although our study does not assess mandibular bone metabolism, it should be recalled that of the bone tissue, bone marrow, periosteum, and surrounding soft tissues, the periosteum plays a key role in the healing of bone fractures (54, 56).

Apoptosis is a key mechanism for maintaining cell homeostasis. It is triggered in two manners: through the external, receptor pathway (induced, inter alia, by high concentration of TNF-α) or internal—mitochondrial pathway (induced, among others, by overproduction of ROS) (57, 58). In the presented study, we demonstrated significantly higher concentration of Bax (pro-apoptotic protein) and Bax/Blc-2 ratio, as well as considerably higher activity of caspase-3 (‘executive' caspase) in the periosteum covering titanium mandible fixations. Thus, titanium implants and their wear debris not only stimulate chronic inflammation (↑IL-1, ↑IL-6, ↑TNF-α, ↑GLU), but also induce apoptosis. This hypothesis is confirmed by the negative correlation between NOX activity and Bcl-2 concentration, as well as positive correlation between Bax concentration and CAS-3 activity, TNF-α concentration and CAS-3 activity, and titanium content and CAS-3 activity (Figure 7). Interestingly, we also observed a significantly higher NO concentration in the periosteum of patients from the study group compared to the control. It is believed that NO entails the activation of cyclooxygenase-2 (COX-2) and intensifies bone resorption processes around the implant by stimulating osteoclasts (59, 60). In the case of chronic inflammations, long-term exposure to NO may cause DNA damage, inhibit its repair and, as a result, activate pro-proliferative signal pathways, and apoptosis (59). Interestingly, *in vitro* studies have shown that titanium particles induce osteoblast apoptosis, leading to inhibition of bone formation around implants (61). Nevertheless, a similar study on the human model is not possible for ethical reasons (inability to take bone tissue from healthy people). Although the physical examination did not reveal any signs of inflammation or osteointegration disorders, it can be assumed that, under unfavorable conditions (stress, disease), ROS-induced inflammation and apoptosis may lead to impaired bone healing as well as cause implant rejection (62).

Despite many advantages, there are certain limitations to our experiment. We evaluated only the selected biomarkers of ROS production, inflammation and apoptosis, and therefore, we cannot fully characterize the nature of these processes. Additionally, as limited by a small amount of research material, we did not assess the consequences of ROS formation, inflammation and apoptosis with respect to bone deformation or remodeling, mineralization, osteoblast activity or dysfunction of vascular endothelium and angiogenesis. However, this is the first study which indicate higher free radical generation as well as increased inflammation and apoptosis in the tissue surrounding titanium mandibular fixations. The strong side of the study is also the restrictive inclusion and exclusion criteria, and the fact that all subjects were at a young age and had similar types of jaw bone fractures. It is also a starting point for future basic and clinical research.

## Conclusions

1. Titanium mandible fixations and their debris products increase the production of pro-inflammatory cytokines and free oxygen radicals in the periosteum covering titanium miniplates and miniscrews.
2. Exposure to Ti6Al4V titanium alloy in the mandibular periosteum induces not only inflammation and ROS overproduction, but also apoptosis. However, no clinical signs have been observed in any of the patients.
3. Further research and clinical observations concerning the influence of titanium fixations on the bone metabolism and the accompanying tissues are necessary.

## Data Availability Statement

The datasets generated for this study are available on request to the corresponding author.

## Ethics Statement

The experiment was conducted in accordance with the Declaration of Helsinki (1964). All patients were informed about the purpose of the study and type of the planned examinations, and signed a conscious consent to participate in the experiment. The protocol of the study was approved by the Bioethics Committee of the Medical University of Bialystok, Poland (permission number R-I-002/3/2-16).

## Author Contributions

We declare that the paper Free radical production, inflammation and apoptosis in patients treated with titanium mandibular fixations—an observational study by JB, MM, BA, JS, MŚ, and AZ has not been published before. The paper is not under consideration for publication anywhere else and it was read and approved by all co-authors. All authors agree to the submission of the manuscript to the *Frontiers in Immunology*. JB conceptualized, interpreted data, wrote the manuscript, and final approval of the version to be published. MM conceptualized, did laboratory determinations, performed statistical analysis, interpreted data, did performance of the graphic part of the manuscript, wrote the manuscript, and final approval of the version to be published. BA, JS, and MŚ did laboratory determinations. MS did laboratory determinations. AZ conceptualized, interpreted data, and final approval of the version to be published.

### Conflict of Interest

The authors declare that the research was conducted in the absence of any commercial or financial relationships that could be construed as a potential conflict of interest.
